# “Planning eye health services in Varamin district, Iran: a cross-sectional study”

**DOI:** 10.1186/s12913-015-0797-0

**Published:** 2015-04-03

**Authors:** Marzieh Katibeh, Karl Blanchet, Shadi Akbarian, Sara Hosseini, Hamid Ahmadieh, Matthew J Burton

**Affiliations:** Ophthalmic Epidemiology Research Centre, Shahid Beheshti University of Medical Sciences, Tehran, Iran; International Centre for Eye Health, London School of Hygiene and Tropical Medicine, London, UK; Ophthalmic Research Centre, Shahid Beheshti University of Medical Sciences, Tehran, Iran

**Keywords:** Community health planning, Vision disorders, Prevention and control

## Abstract

**Background:**

A recent survey of avoidable blindness in Varamin District, Iran, identified moderately high levels of visual impairment (10%) and blindness (1.5%) in people >50 years. This study aimed to define current provision, identify gaps and suggest practical solutions for improving eye health services in this area.

**Methods:**

The World Health Organization (WHO) framework for analyzing health systems has several key components: service delivery, health workforce, information system, medical products and technologies, financing, and governance. We used this structure to investigate the strengths and weaknesses of the eye health system in Varamin. All public and private eye care facilities and a random selection of primary health care (PHC) units were assessed using semi-structured researcher-administered questionnaires.

**Results:**

Varamin has 16 ophthalmic clinics, including two secondary hospitals that provide cataract surgery. There were ten ophthalmologists (1:68,000 population), two ophthalmic nurses and five optometrists working in Varamin district. There were no eye care social or community workers, ophthalmic counsellors, low vision rehabilitation staff. Although the Vision 2020 target for ophthalmologists has been met, numbers of other eye care staff were insufficient. The majority of patients travel to Tehran for surgery. The recent survey identified cataract as the leading cause of blindness, despite the availability of surgical services in the district and high health insurance coverage. Poor awareness is a major barrier. No units had a written blindness prevention plan, formal referral pathways or sufficient eye health promotion activities. Only one of the PHC units referred people with diabetes for retinal examination. There is partial integration between eye care services and the general health system particularly for prevention of childhood blindness: chemo-prophylaxis for *ophthalmia neonatorum*, school vision tests, measles immunization and Vitamin A supplementation.

**Conclusions:**

This analysis demonstrated the need for better integration between eye care services and the general health system, local planning for prevention of blindness, an information system, a better staff mix and health education to increase community awareness and service uptake. There is the capacity to deliver far more surgery locally. All aspects of a health system need to be developed to deliver comprehensive and efficient eye care.

## Background

“Vision 2020: The Right to Sight” is a global initiative for the prevention of blindness that was launched in 1999 by the International Agency for Prevention of Blindness and the World Health Organization (WHO) [1]. Vision 2020 promotes the development of health systems to deliver integrated, sustainable, affordable, accessible and equitable eye care services through district level structures [2]. The approach that each country takes to implement a prevention of blindness program depends on existing human resources, infrastructure and population needs [3]. Recently the WHO Global Action Plan (2014–2019) has provided an updated approach with increased emphasis on the health systems approach and integration of eye care into the general health system [4]. To provide a rational basis to improve services the Action Plan highlights the need for a comprehensive assessment of available resources and current service gaps [4]. This is usually followed by the development and implementation of a locally appropriate plan to improve the quality and quantity of both the clinical and non‐clinical aspects of care, which addresses local barriers [5]. Some key indicators in monitoring progress of the Global Action Plan are number of eye care professionals per million population, the cataract surgical rate and cataract surgical coverage [4].

The vast majority of blindness in Iran, in common with many other countries and the global estimates, is due to treatable or preventable causes, such as cataract and refractive errors that can be alleviated by developing preventive or therapeutic strategies [6-10]. We recently conducted a Rapid Assessment of Avoidable Blindness (RAAB) survey in Varamin District of Iran. It is therefore possible to relate population eye care needs and current resources and services in the same district [8]. The Varamin RAAB survey found that about 10% of over 50 year olds suffer from bilateral visual impairment (VA <6/18) and 1.5% of people in this age group are blind (VA <3/60) [8]. Cataract was the main cause, accounting for 47.5% of severe visual impairment and 31.7% of blindness; it remains the largest eye care need in this district. Uncorrected refractive error is responsible for almost 10% of severe visual impairment (SVI: 3/60 ≤ VA < 6/60) and 50% of moderate visual impairment (MVI: 6/60 ≤ VA < 6/18). A similar pattern has been reported from other countries in the Middle East. For instance, cataract was the leading cause of blindness in Pakistan (51.5%) and Saudi Arabia (41%) [11,12]. These rates are higher than those of high-income countries, where the proportion of all cause blindness due to cataract is less than 5% [13,14]. Diabetes is a matter of particular concern in Iran. Population-based studies found 14% of urban adults to have diabetes and the prevalence of diabetic retinopathy to be 37% [15,16].

The aim of this study was to define current provision, identify gaps and suggest practical solutions for improving eye health services in the Varamin District, in line with the principles laid out in the WHO Global Action Plan (2014–2019).

## Methods

This study is a cross-sectional descriptive survey of all public and private eye care facilities within Varamin District, Iran. The study received ethical approval from the Ethics Committees of Shahid Beheshti University of Medical Sciences and the London School of Hygiene and Tropical Medicine. All potential participants were asked to read the study information sheet and document their willingness to participate by signing a consent form. Data collection took place between June and August 2013.

### Setting

**Figure 1:**
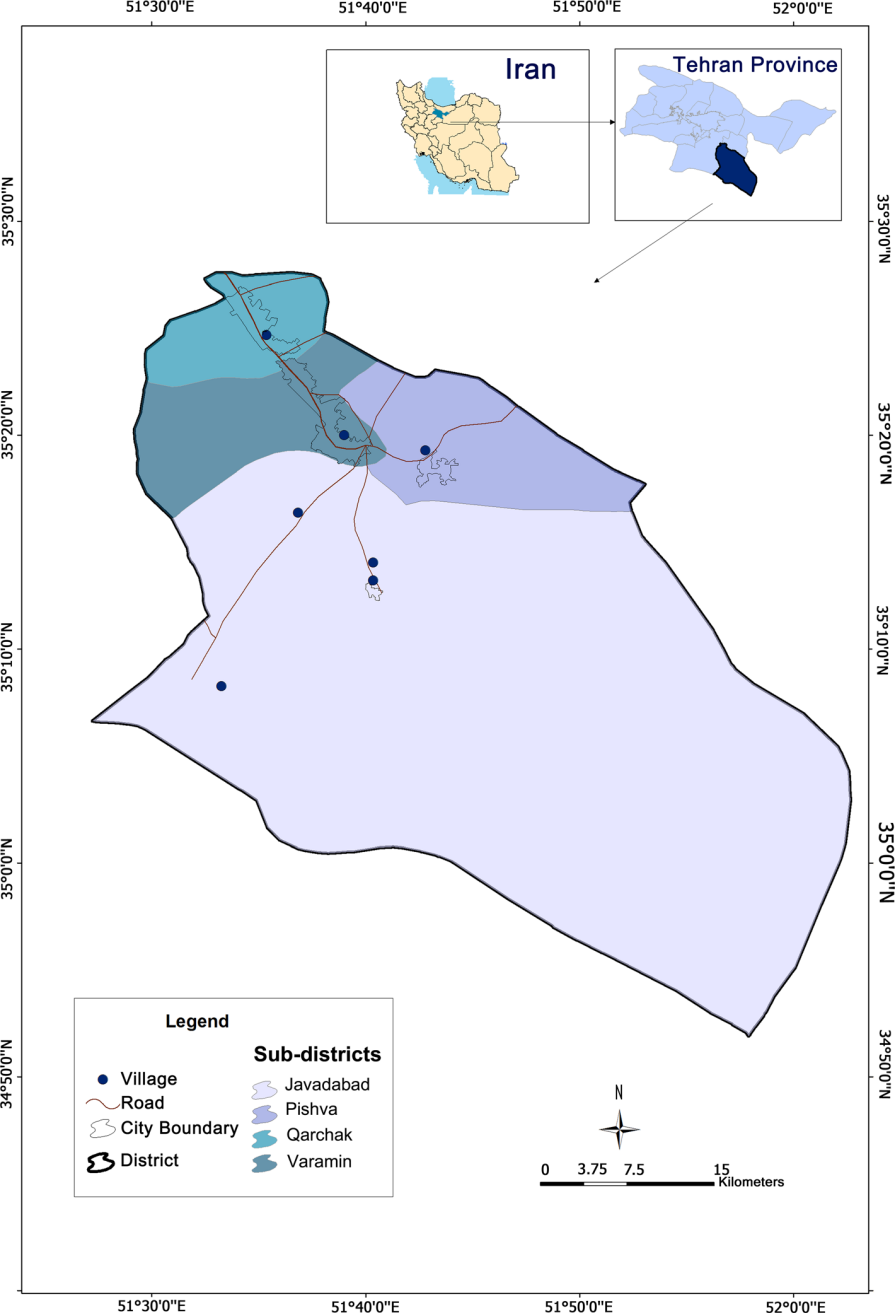
Varamin district map and population. The map only shows larger villages.

Varamin is one of of about 425 administrative districts of Iran. It is located 27 miles from Tehran, the capital city and is one of the districts of Tehran province. According to the 2011 National Census, Varamin had a population of 542,832 [17]. About 80% of the population live in urban areas; primarily the four cities/towns of Varamin, Pishva, Gharghack and Javadabad. The remaining 20% of the population live in villages distributed widely across the district (Figure 1).

### Sampling frame

All urban and rural ophthalmic and Primary Health Care (PHC) units within the district were identified from the latest registration list of the Health Care Department of Varamin District as the sampling frame.

First, all ophthalmic units (private and public) located within the district were included in the study sample. Ophthalmic units were defined as a facility where at least one ophthalmologist or one optometrist work and deliver outpatient consultations, refraction, spectacle dispensing or surgical services.

Second, six PHC units were selected. They were defined as a facility where general health care services were provided by health workers and/or general physicians: mother and child care, vaccinations, health education, family planning and primary treatments. In the current study, six PHC units were randomly chosen out of a total of 56 PHC units. The PHC units in Iran are of several types according to the health system network [18]. The 56 PHC units in Varamin included three categories: Rural Health Houses (26 units), Urban Health Posts (15 units), and Urban Health Centers (15 units). To have a representative sample of PHC units we randomly selected two units for detailed study from each of these three categories.

### Data collection

To evaluate the current status of the district eye care system, we used the six Health System Building Blocks introduced by WHO: (1) service delivery, (2) health workforce, (3) information, (4) medical products, vaccines and technologies, (5) financing, and (6) leadership and governance [19]. This approach has been adapted specifically for the assessment of eye health systems. [20].

Two sets of questionnaires were developed and used for data collections in this study. These were semi-structured, pretested and researcher-administered. One questionnaire was used for data collection in all ophthalmic units and the second tool was used for the six PHC facilities.

All facilities in the study were visited in person by the same study investigator. The facility’s resources were evaluated by direct observation and by interviewing at least one well-informed staff member (the facility manager or an eye care professional), who had up-to-date and adequate information about the facility. The overall condition of the buildings was determined as good, fair or poor, based on several factors including cleanliness, painting, temperature, ventilation, lighting, having enough amenities and space. The maintenance process was assessed based on quality and speed of repairs.

To assess the strengths and weaknesses of primary eye care services, a number of specific activities were selected from the WHO’s strategy on blindness and assessed at the six PHC units [3]. The WHO strategy includes several preventive and clinical activities that can be done at the primary health care level to manage conditions that may lead to visual loss including: (1) implementation of school vision screening programmes, (2) early detection and management of common blinding conditions (cataract/white pupil and diabetic retinopathy screening), (3) treatment and/or referral activities for red eye, low vision and eye trauma, (4) use of Snellen chart for visual acuity [VA], (5) presence of dedicated eye health staff, such as community eye health workers or optometrists, (6) availability of essential eye medicines and equipment, (7) written local manuals and guidelines for eye health, (8) implementation of community-based eye care educational programmes, (9) Vitamin A distribution, and (10) measles vaccination. We excluded several conditions which can potentially be managed or followed-up at PHC level either because they are uncommon in Iran (Onchocerciasis), less serious (pterygium, allergic conjunctivitis), or more complicated (glaucoma). We investigated how three common ophthalmic emergency presentations (acute red eye, acute vision loss and eye trauma) and two common chronic eye diseases (cataract and diabetic retinopathy) were managed or referred by PHC workers at the selected health units. The assessment of integration of eye health services within the general health system at PHC level was based on the presence of above mentioned essential equipment, medicines, programmes and human resources in the PHC units.

### Statistical analysis

Data were managed in MS Access and exported to MS Excel and SPSS (V 17.0) for analysis. Geographical addresses of the units visited were entered to Google Map and the coordinates were exported to ArcMap 10.1 to generate maps. We used the Kruskal-Wallis test to compare different eye units in terms of waiting time.

## Results

### Eye units

There were 16 eye care facilities: ten private clinics, two secondary hospitals and four charity clinics, all located in urban areas.

### Eye health workforce

Ten ophthalmologists and five optometrists worked in these 16 eye units. None of the eye units employed a full-time ophthalmologist; therefore, most ophthalmologists worked in more than one facility inside the district. Taking into account the number of working days per week, the total number of Full Time Equivalent (FTE) ophthalmologists was eight; therefore, the ophthalmologist to population ratio was 1:68,000.

There were 2 ophthalmic nurses, who both worked for the district public hospital. The other hospital had no dedicated ophthalmic nurse; therefore, in-patient services and surgery were supported by general nurses who had rotational shifts between all units. There were no dedicated eye care social or community workers, ophthalmic counsellors, low vision rehabilitation staff or any other cadres dedicated to eye health within the district. These ophthalmic nurses were not involved in outreach activities or primary-level eye care services. Their main role was assisting ophthalmologists in delivering in-patient and out-patient services to patients who were referred to the district hospitals.

The type and number of eye care workers in comparison to the Vision 2020 targets are presented in Table 1 [21]. The Vision 2020 target for the number of ophthalmologists has been met but the number of ophthalmic nurses and community or social health workers is currently insufficient.

**Table 1:** **Comparison of eye care personnel working in Varamin District and the Vision 2020 recommendation**

	**Number**	**Vision 2020 target**	Number needed*	**Deficit (n)**	**Need met (%)**
Ophthalmologist	8**	1:100,000	5	0	160%
Optometrist	5	1:50,000	10	5	50%
Ophthalmic nurses	2	1:25,000	20	18	10%
Community Eye Worker	0	1:10,000	50	50	0%

*The numbers are calculated for the Varamin district with an approximate population of 500,000.

**Full time equivalent ophthalmologist.

### Infrastructure and equipment

Most eye care units were equipped with essential diagnostic and refraction equipment including slit lamps (87.0%), direct ophthalmoscopes (75.0%), VA charts (100%), retinoscopes (87.5%), lensometer (87.5%) and trial lens sets and frames (100%) and 93% of eye units had an associated optical shop in the same place for dispensing spectacles (Tables 2 and 3). Only the two district hospitals had the necessary equipment for cataract and some other eye surgeries. There were no laser treatment devices (YAG or Argon) in any of the units in Varamin District; all patients were referred to hospitals in Tehran for treatment where laser therapy and vitreoretinal surgery are available in tertiary hospitals.

**Table 2:** **Available facilities for eye care in the Varamin District**

	**Private clinics (N = 10)**		**Charity clinics (N = 4)**		**Public Hospitals (N = 2)**		**Total (N = 16)**
Infrastructures	D	S	D	S	D	S	D + S
Examination stations	11	0	4	0	2	0	17 (93.7%)
Operating room	0	0	0	0	1	1	2 (12.5%)
Ophthalmic ward	0	0	0	0	1	0	1 (6.25%)
In-patient bed	0	0	0	0	0	8	8 (12.5%)
Vehicle	0	0	0	0	0	2	2 (6.2%)
Optic shop*	11	0	4	0	1	0	16 (93.7%)

In parentheses are the proportion of eye care units that had at least one on the infrastructure items at the facility.

D: dedicated.

S: shared with other hospital departments.

*there are more optical shops in the district but only those that are linked to an eye units are shown in this table.

**Table 3:** **The number of items of equipment and the number of eye units with that equipment**

**Clinic Equipment**	**Private clinics (N = 10)**		**Charity clinics (N = 4)**		**Public hospitals (N = 2)**		**Total (N = 16)**	
	**Equip. (n)**	**Unit (n, %)**	**Equip. (n)**	**Unit (n, %)**	**Equip. (n)**	**Unit (n, %)**	**Equip. (n)**	**Unit (n, %)**
Slit Lamps	10	8 (80%)	4	4 (100%)	2	2 (100%)	16	14 (87.0%)
Slit Lamp Lenses	15	7 (70%)	6	3 (75%)	6	2 (100%)	27	12 (75.0%)
Goniolens	7	7 (70%)	2	2 (50%)	1	1 (50%)	10	10 (62.5%)
Direct Ophthalmoscopes	9	8 (80%)	2	2 (50%)	2	2 (100%)	13	12 (75.0%)
Indirect Ophthalmoscopes	6	6 (60%)	1	1 (25%)	2	2 (100%)	9	9 (56.2%)
Diode Laser (Photocoagulation, CPC)	0	0	0	0	0	0	0	0
Photocoagulation Laser	0	0	0	0	0	0	0	0
NdYAG Laser	0	0	0	0	0	0	0	0
Fundus Cameras	0	0	0	0	0	0	0	0
Retinoscopes	10	9 (90%)	3	3 (75%)	2	2 (100%)	15	14 (87.5%)
Tonometers	8	7 (70%)	2	2 (50%)	2	2 (100%)	12	11 (68.7%)
VA testing charts	11	10 (100%)	4	4 (100%)	2	2 (100%)	17	16 (100%)
Trial Lens sets and frame	11	10 (100%)	4	4 (100%)	2	2 (100%)	17	16 (100%)
Visual Field Analysers	0	0	1	1 (25%)	0	0	1	1 (6.2%)
A Scan for IOL calculation	0	0	0	0	2	2 (100%)	2	2 (12.5%)
B Scan ultrasound	0	0	0	0	1	1 (50%)	1	1 (6.2%)
Pachymeters	0	0	0	0	0	0	0	0
Auto-Keratometer	1	1 (10%)	1	1 (25%)	1	1 (50%)	3	3 (18.7%)
Refractor Keratometer	12	10 (100%)	3	3 (75%)	2	2 (100%)	17	15 (93.7%)
Keratometers	1	1 (10%)	0	0	1	1 (50%)	2	2 (12.5%)
Orbscan	0	0	0	0	0	0	0	0
Topography	0	0	0	0	0	0	0	0
Lensometer	8	8 (80%)	4	4 (100%)	2	2 (100%)	14	14 (87.5%)
Color vision test (titmus test)	1	1 (10%)	0	0	0	0	1	1 (6.2%)

Equip. (n): the total sum of equipment in each row available in Varamin district, some units may have more than 1 piece of equipment.

Unit (n. %): the number and proportion of eye units that have at least 1 piece of equipment.

**Figure 2:**
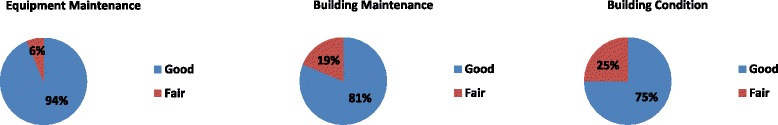
The building condition and maintenance process of the 16 eye units in the Varamin.

There was only one dedicated operating theatre for ophthalmic surgery in Varamin District in one of the hospitals. In the other district hospital, eye surgery was performed in an operating theatre shared with other departments. Beside the local hospitals that were equipped for cataract surgery, other units, including 10 private and 4 charity clinics referred people for eye surgery to either Tehran or to the two local hospitals. All units had constant access to electricity. Building conditions and maintenance support were reported as acceptable in all units (Figure 2). Although there was just one ophthalmic equipment maintenance technician who worked in one of the local hospitals, local medical device companies had after-sales services and almost all eye units had prompt access to external maintenance technicians.

### Service delivery

The total number of ophthalmology consultations and procedures delivered through the two hospitals were recorded accurately, as these were linked into the hospitals’ financial and health insurance data management systems for payment. In contrast, precise data on service delivery by the private and charity clinics were probably less reliable as financial data management systems were not developed to the same level and the staffs at these units were of the view that the data may not be reliable. The data presented here are the estimated numbers of different services delivered during the month prior to the assessment. The number of different services by facility type is presented in Table 4.

**Table 4:** **The number of delivered eye services by facility type in Varamin District**

	**Facility type (monthly)**						**Total**			
	**Private clinics (n = 10)**		**Hospitals (n = 2)**		**Charity Clinics (n = 4)**		**Monthly**		**Yearly** ^**§**^	
	**L**	**R**	**L**	**R**	**L**	**R**	**L**	**R**	**L**	**R**
Eye exam	1920	133	450	75	420	0	2790	208	30690	2288
Refraction	1708	0	420	0	390	0	2518	0	27698	0
Dispensing	889	0	300	0	197	0	1386	0	15246	0
Low vision	0	12	0	2	0	6	0	20	0	220
Cataract surgery	0	71	58	0	0	93	58	164	638	1804
Pediatric surgeries	0	20	0	0	0	8	0	28	0	308
Other surgeries*	0	91	6	2	0	37	6	130	66	1430
DR screening	132	95	20	4	24	8	176	107	1936	1177
Yag laser	0	34	0	3	0	4	0	41	0	451
Retinal laser	0	42	0	4	0	1	0	47	0	517

^§^11 working months and one holiday month.

L: delivered in the local facilities.

R: referred to Tehran.

*included chalazion, vitreo-retinal surgeries, dacryocystorhinostomy (DCR), refractive surgeries, pterygium, glaucoma.

If we assume that all those who were referred to Tehran for cataract surgery went and received treatment, the Varamin District Cataract Surgical Rate (CSR) was estimated to be in 4884 surgeries/million population/year (July 2012- June 2013). If we only consider the number of cataract surgeries performed by the two district hospitals, the CSR was 1276/million population/year. Dividing the number of annual eye surgeries by the number of full time equivalent ophthalmologists who worked in this district, there were about 305 cataract surgeries/ophthalmologist and 80 cataract surgeries per FTE ophthalmologist in the local facilities were delivered between July 2012 and June 2013.

There was no waiting list for the first outpatient appointment at ten private units. In the other six units, patients were usually seen within 7 days. For those requiring cataract surgery, the mean wait for surgery was 7.1 days (SD ± 6.1) after the first outpatient appointment. There was no association between the different types of units and the numbers of days spent waiting for cataract surgery (p = 0.34) and the number of patients on the waiting list (p = 0.45).

### Leadership and governance

Ten (62,5%) of the eye care units were established and equipped by private ophthalmologists or optometrists. The local community participated in supporting the four charity clinics, which were built and run by local individual philanthropists.

Except for the charity clinics, units were managed by a medical professional including ophthalmologists, medical doctors or optometrists. The managers of the charity clinics did not have any medical training. Both hospitals had eye unit managers who were responsible for organizing in- and out-patients services. These managers were not responsible for district level planning for eye health and prevention of blindness. The Varamin District Health Care Department inspected local eye units one to four times a year, excluding the hospital that performed the largest number of eye surgeries in Varamin District. In these visits the qualification and certificates of workforces, building condition, medical equipment and existence of any illegal activities were checked.

One hospital had a formal plan for patient satisfaction and safety; the other eye units had no formal plans. Amongst the latter, six units had unwritten and informal plans for improving patients flow or building conditions. However, there were no specific plans for improving quality, productivity, efficiency, or prevention of blindness. There were no written guidelines for where patients should be referred to receive surgical treatment.

### Information system

Eye units were not asked by the District Health Authority to report vision-related disorders, procedures or indicators such as the number of blind people, or number of surgeries performed. There were no formal links between the different units, the supervisory organizations or primary health units in terms of sharing information or reporting the number and quality of delivered services. There were no formal mechanisms for referring patients between units. It was reported that in general patients were given verbal advice about where to access further services and written referral correspondence was generally not provided.

### Finance

The price of cataract surgery and other services are summarized in Table 5. Although social and commercial insurance schemes covered more than 90% of people in Iran, in the current study, eye health providers in most eye units mentioned that patients had to pay for non-surgical services including outpatient visits, refraction and spectacle provision. In the two hospitals, people with some kinds of state insurance schemes could get access to free or subsidized surgical services. The tariffs of cataract surgery ranged from about 4,000,000 IRR in some charity clinics to 40,000,000 IRR in some private units. In local public hospitals, the cost of cataract surgery ranged between 5,000,000 IRR to 18,866,000 IRR due to different IOLs used, different surgical procedures and admission duration.

**Table 5:** **The price of ophthalmic services in Varamin district**

	**Mean**		**Minimum**		**Maximum**	
	**IRR**	GBP*	**IRR**	**GBP**	**IRR**	**GBP**
Cataract Surgery	14000000	280	4000000	80	40000000	800
IOL	2500000	50	500000	10	4500000	90
Ophthalmologist Visit	180000	3.6	130000	2.6	200000	4
Optometrist Visit	80000	1.6	40000	0.8	100000	2
Spectacles	700000	14	300000	6	3500000	70

*Converts are based on the average market prices in 2013.

Nine eye units (56.3%) including all charity clinics, four private clinics and one hospital had free or subsidized services for patients who could not afford to pay. The subsidies were provided by individual donors or by the government. However, the extent of this subsidized provision could not be accurately quantified.

#### General health units

There were 56 public health facilities including the District headquarters, 15 urban posts, 26 rural health units and 15 urban health units located inside Varamin district. Table 6 shows a summary of primary eye care activities in the six assessed PHC units.

**Table 6:** **Summary of primary eye care activities in the six PHC units in Varamin**

	**Number of units**	**Proportion**	**Details**
High level staff with some formal training in eye care	2	33.3%	A full-time GP worked in each unit and a part-time optometrist worked one day a week in one of these two units
Mid-level eye health staff	-	-	Such as ophthalmic nurse or health workers trained in eye care
Community eye care workers	-	-	
Written plan for eye health	-	-	
Written guidelines for eye health and prevention of blindness	2	33.3%	The 2 units had a booklet called “Healthy Infant” covering major eye health issues for children
Snellen VA charts	5	83.3%	Snellen chart was used at least once a week in 4; in the other two units a chart was never or only rarely used
Ophthalmoscope	1	16.6%	
Measles vaccination	6	100%	
Vitamin A supplementation	6	100%	
Red eye treatment	2	33.3%	
Delivering primary eye care for eye trauma	1	16.6%	
Screening programme for diabetic retinopathy	1	16.6%	
Early detection of cataract	2	33.3%	In these units newborns and infants were examined for white pupil
Formal referral pathways to the secondary and tertiary levels	-	-	
Eye health promotion for the community	-	-	

None of PHC units had mid-level eye health staff, such as an ophthalmic nurse or health workers trained in eye care. There were no community eye care workers or ophthalmic nurses at the community level. In the urban PHC units, general physicians provided treatment and referrals for common eye problems but were not involved in preventive eye care activities or active case detection of eye disease which was expected from community eye care staff. Therefore, in terms of service delivery, the eye health system staff did not work outside the context of specialist eye units (either in the secondary hospitals or the specialist clinics). According to the staff who were interviewed, there were no clearly defined systems for referring people with eye problems and no mechanism to ascertain whether they received treatment, which suggests a lack of integration of eye care into the general health system in terms of referral system.

### Eye health education and promotion

None of the six PHC units that were assessed had a written plan for eye health, prevention of blindness, eye health education or treatment guidelines for adults, suggesting that there was no systematic basis for their integration. Health workers at two units had received a booklet called “Healthy Infant”, which addressed prevention of childhood blindness as a part of their training course: *ophthalmia neonatorum*, white pupil, amblyopia and red eye.

### Essential equipment and medicines

Five PHC units had Snellen VA charts for measuring visual acuity and the health workers had been trained to test vision. The VA test is an important part of the eye examination for many eye problems that should be managed at and/or referred from PHC units including eye trauma, visual loss or red eye. In four units, the Snellen chart was used at least once a week whereas in the other two units a chart was never or only rarely used. Just one unit had an ophthalmoscope. Medical Officers or general physicians based at primary health care facilities are supposed to be able to examine the optic disc and macula with a direct ophthalmoscope.

All units reported 100% coverage for measles vaccination, chemo-prophylaxis for *ophthalmia neonatorum* and Vitamin A supplementation. Measles or MMR vaccines were given to all infants twice, at 9 and 15 months. Vitamin A was also given for free to all children under two years who were brought to the PHC unit for health care or immunization. This supplementation began 15 days after birth and continues until the second birthday. In addition, Vitamin A was given to children with diarrhoea or malnutrition and to mothers after delivery. The health centres distributed free generic Vitamin A + D drops as part of the mother-child PHC programme. Therefore, integration of essential medicines related to childhood eye health was achieved in Varamin District.

### Disease control

At four PHC units, patients with red eye were initially referred to an ophthalmologist. There were various referral patterns; some units guided people directly to a local private or charity clinic and some sent people to another health unit with a general physician. In the units with a general physician, local antibiotics were prescribed for red eye. However, the treatment or referral processes were not uniform among health units or based on formal written guidelines.

For acute vision loss or eye trauma, a VA test would be taken at five units and patients directed to visit a private clinic or public eye unit. The VA was usually written in a referral letter. No units had written guidelines for managing eye trauma or vision loss. The PHC units referred patients immediately without delivering any primary eye care before sending patients with eye trauma to a suitable centre for treatment.

Five units had no formal or informal screening programme for diabetic retinopathy. In one unit, patients with diabetes were recorded and followed in terms of blood sugar control and prevention of diabetic complications. In that PHC unit, all people with diabetes were annually referred to an eye clinic for fundus examination.

There was no formal plan, particularly at the community level, to detect and/or refer elderly adults with cataract (white pupil) to an ophthalmologist. Two units had screening programs for infant cataract. However, the referral process was not organized or well documented. Overall, in terms of control of blinding eye conditions and common eye disorders eye care services were not integrated into the general health system.

### School vision tests

All health units were involved in school VA screening programmes and the coverage of this activity was reported as 100% of school aged children. Vision tests are provided free of charge annually in all primary, secondary and high school children. Health workers from five PHC units measured VA of school children; in one PHC unit this was done by a school nurse. In all units, after detecting children with a vision problem, parents or care-givers were informed and children were referred to an optometrist or ophthalmologist.

## Discussion

This is the first study on district level ophthalmic resources and eye care services in Iran and their degree of integration into the general health system. Six key components of the eye care system and some aspects of PHC system were evaluated to define the current provision, identify gaps and suggest practical solutions for improving eye health services in this area.

### Resources and challenges of eye care system

Despite the high number of ophthalmologists working in Varamin district (Table 1), which exceeds the minimum Vision 2020 recommendation [21], and two district level hospitals that are equipped to perform cataract surgery, cataract was still the leading cause of blindness.

In many low and middle income countries, more than 50% of blindness is due to untreated cataract. This is often attributable to the limited number of human and other resources [22-24]. In addition to resources, some other barriers need to be overcome including the lack of education [25] and transportation limitation [26]. Varamin district does not appear to have limited eye care staff or infrastructure, nor does the direct cost of cataract surgery seem to be a serious barrier, however, on the basis of the RAAB survey findings, we believe the community’s cataract surgical needs are still currently not being met.

In a recent population-based survey of cataract in Varamin, we found that the main barrier to cataract surgery reported by patients who were blind from cataract was a lack of awareness of treatment [27]. This highlights the importance and need for improved health education and health promotion activities in this district. It is difficult to accurately estimate the total CSR for Varamin as we estimate that perhaps three-quarters receive their treatment in Tehran. If all those referred to the capital for surgery actually received treatment the CSR would be almost 5,000. However, the “locally-performed CSR” is much more modest at around 1,300, which is comparable with the national CSR reported for the whole country (1,331 in 2005) [28].

The WHO estimates that an ophthalmologist can be expected to perform 1,000-2,000 cataract operations per year, if they are well supported and patients present for surgery [29]. Therefore, the cataract surgery need of the population of Varamin District could be met by the two district hospitals with sufficient human recourses and good management. If one applies the WHO potential productivity value of 1,000 cases per year, the ophthalmologists working in Varamin District might collectively be expected to be able to perform upwards of 8,000 surgeries each year. Therefore, the current number of about 2,442 (both local and referred cases) cataract surgeries per year represents a low productivity compared to the potential of this group.

Although the ratio of ophthalmologists and other support staff to the population being served are key Vision 2020 Initiative and Global Action Plan indicators, the geographical distribution of human resources is also very important [2,30,31]. Often the clinicians are concentrated in larger urban centres, with rural populations relatively underserved. In Nigeria for example, although the overall number of eye care workers were sufficient to serve the population in Enugu Urban and Enugu State, an uneven geographical distribution of this workforce created a major barrier to uptake of eye care services [32]. Similarly, in Southern Ethiopia the provision of cataract surgery services is concentrated in a few urban centres, with much of the population having no access to services [33].

The 2011 National Census estimated that 75 million people lived in Iran [17]. According to the Iranian Society of Ophthalmology, there are more than 1,500 ophthalmologists in the country. However, the distribution and effectiveness of these specialists has not been addressed. The large number of ophthalmologists working in Varamin District is not an entirely positive finding as this reflects a wider issue faced in Iran of ophthalmologists tending to work in and around Tehran and other large cities. Other parts of the country are less well served: for example, the prevalence of blindness in two remote southern provinces Sistan-va-Baluchestan and Khuzestan is 2–4 times more than Tehran [7,9,10]. Secondly, our impression is that unhelpful competition may have developed between the private units, focusing efforts on more lucrative activities including early cataract surgeries and optical shops, leaving other issues less well addressed. Providing reasonable incentives and good facilities for professional and support staff who serve in remote areas, establishing rotational outreach visits, and finally strengthening district level eye care planning are some of the solutions for more equal human resource distribution. In addition, a situation analysis to understand the location preferences of health workers can form the basis for interventions to promote greater acceptance of less desirable placements [34].

The eye care workforce in Varamin district lacks a suitable staff-mix, compared to that recommended by the Vision 2020 Initiative [35]. There were no community level workers and only very few nurses with ophthalmic training; there is a need for a better mix of professional, mid-level and community-level staff to increase access, demand and efficiency. Instead of simply targeting an overall national increase in ophthalmologists and eye beds, a region specific approach should be taken, to promote a more equitable distribution of the available resources [2,36]. Although the latest WHO strategy for prevention of blindness for 2014–2019 encourages member states to conduct local planning and target setting [4] in this situation analysis we followed the earlier Vision 2020 framework because the universal eye care approach has not yet been implemented in Iran and provides some bench marks for comparison.

Almost all units were visited by the local health authorities at least once a year and most units kept medical records. Nevertheless, the information system for eye care needs strengthening; the number of patients, the type and number of different services delivered were not reported and the local units had not received feedback.

The number of people from Varamin District who were identified as having low-vision needs and who were referred to low-vision services was about 220 per year (population ≈ 0.5 million), which is higher than that anticipated by the Vision 2020 Initiative (200 low vision treatments/year/million population) [2]. However, there was no place within Varamin District where people could get rehabilitation services or buy low vision devices. It is not known whether those referred to Tehran for low-vision services were able to access or afford these.

The eye health system in Varamin district had several positive aspects; the time to wait for ophthalmic consultations and the time to wait for cataract surgery was at most 7 days. Some ophthalmologists and/or optometrist were serving in local charities, running eye care facilities. This is a good sign of the broader society engaging with eye health. According to our recent study, the outcome of cataract surgery in Varamin district was desirable (post operative visual acuity ≥ 6/18) in 71.9% of operated eyes [27]. However, cataract outcome data was not routinely collected; a lack of regular cataract audit is a weakness of the eye care system in this district.

### General health system challenges

To achieve better population eye heath, Vision 2020 and the Global Action Plan promote the integration of eye care services into the general health system [3,4,37]. Health services would ideally be available as close as possible to where people live and be affordable [38].

In Varamin District, some primary eye care services were integrated well at PHC level. These included the management of *ophthalmia neonatorum* and Vitamin A supplementation, which were integrated into the Maternal and Child Health Program and measles vaccination, which is integrated into the Immunization Program. There is a school health program in Iran that includes school eye examinations and some other PHC services for 6–18 years old children. The coverage of school eye exams and measles vaccination was reported as 100% and the majority of eye units had a vitamin A supplementation program. The number of blind children in Iran due to corneal blindness and malnutrition is very low and similar to developed country levels, indicating that the current programme for the prevention of childhood blindness is working well [39].

For other conditions, there is currently little or no integration of eye care into the general health system in Varamin. This needs to be addressed. The general health services have considerable potential to address avoidable blindness through health education, preventive treatment of important eye diseases, recognition of symptoms and early referral, screening programmes, and rehabilitation services for blind or visually impaired people [3,5].

Our earlier work in Varamin district found the main barrier to cataract surgery among untreated people was being “unaware of treatment”. Most people with untreated cataract did not know their problem was treatable or that treatment was available through local eye care services [27]. However, there are no systems to identify people who are unaware they have a treatable visual problem. In addition, only one out of six PHC units looking after people with diabetes had a system in place for diabetic retinopathy screening. We found that there is no training provided on eye care to general health workers and there are no written clinical guidelines for common conditions.

According to the staff that we interviewed, there is limited interaction between primary, secondary and tertiary levels in terms of information flow, case finding, referrals, training, feedback and supervision. Patients can obtain services without having referral notes. Therefore, the number of patients attending the tertiary hospitals in Tehran may be unnecessarily high as many could be managed at the primary and secondary level. A strengthened referral pathway that creates a strong link between different levels of the health system is required to achieve the Vision 2020 goals [40]. In addition, it is recommended that current PHC activities could be examined to determine whether they have addressed the eye care challenges in this district.

Fortunately, in Iran more than 90% of people have health insurance, which generally covers cataract and other essential surgeries [18]. There are several public and private agencies managing health financing, possibly reducing inefficiency and raising costs [41]. Some people may not be aware that they can reduce the costs by obtaining treatment within Varamin District rather than in Tehran, where they incur additional indirect costs for travel and accommodation and possibly longer waiting times at the tertiary referral hospitals.

The Vision 2020 Initiative promotes integrated, sustainable, equitable and excellent services for all people who need eye care [21]. The latest WHO Action Plan for prevention of blindness, has introduced additional principles, including universal access to eye care, equity, human rights, evidence-based practice, a life course approach, and empowerment of people with blindness and visual impairment [4]. To achieve these aims in Varamin, we need district-level Vision 2020 plans which are integrated into the health system, with PHC level treatment and referral protocols [37].

This study has some limitations that could be explored further in future studies. We were only able to quantify existing services and facilities like school screening programme, essential medicines, equipment and educational materials. The assessment of the quality and efficiency of these services was beyond the objectives and scope of the current study. As a cross-sectional study, it was not possible to determine the proportion of patients referred from Varamin eye units and PHC units to Tehran or other facilities who actually went and received treatment. Finally, the data on the services delivered by the private units may not be as complete as that available from the public units, potentially underestimating the activity.

## Conclusion

The development of an effective eye care programme to prevent or treat avoidable causes of blindness needs a package of measures that link the different service levels and integrates eye care into the wider health system. An analysis of the eye care system in Varamin district, applying the WHO’s six health system building blocks has helped to identify several key areas where the system is likely to benefit from strengthening.

## References

[1] Pararajasegaram R (1999). VISION 2020-the right to sight: from strategies to action. Am J Ophthalmol.

[2] Cook C, Qureshi B (2005). VISION 2020 at the district level. Community Eye Healt.

[3] World Health Organization (1997). Strategies for the prevention of blindness in national programmes-A Primary Health Care Approach.

[4] World Health Organization (2013). Action plan for the prevention of avoidable blindness and visual impairment 2014–2019. Towards universal eye health: A global action plan 2014 -2019.

[5] Hubley J, Gilbert C (2006). Eye health promotion and the prevention of blindness in developing countries: critical issues. Br J Ophthalmol.

[6] Stevens GA, White RA, Flaxman SR, Price H, Jonas JB, Keeffe J (2013). Global prevalence of vision impairment and blindness: magnitude and temporal trends, 1990–2010. Ophthalmology.

[7] Shahriari HA, Izadi S, Rouhani MR, Ghasemzadeh F, Maleki AR (2007). Prevalence and causes of visual impairment and blindness in Sistan-va-Baluchestan Province, Iran: Zahedan Eye Study. Br J Ophthalmol.

[8] Rajavi Z, Katibeh M, Ziaei H, Fardesmaeilpour N, Sehat M, Ahmadieh H (2011). Rapid assessment of avoidable blindness in Iran. Ophthalmology.

[9] Fotouhi A, Hashemi H, Mohammad K, Jalali KH (2004). The prevalence and causes of visual impairment in Tehran: the Tehran Eye Study. Br J Ophthalmol.

[10] Feghi M, Khataminia G, Ziaei H, Mahmood L (2009). Prevalence and causes of blindness and low vision in Khuzestan province, Iran. J Ophthalmic Vis Res.

[11] Dineen B, Bourne RRA, Jadoon Z, Shah SP, Khan MA, Foster A (2007). Causes of blindness and visual impairment in Pakistan. The Pakistan national blindness and visual impairment survey. Br J Ophthalmol.

[12] Al Ghamdi AH, Rabiu M, Hajar S, Yorston D, Kuper H, Polack S (2012). Rapid assessment of avoidable blindness and diabetic retinopathy in Taif Saudi Arabia. Br J Ophthalmol.

[13] Kocur I, Resnikoff S (2002). Visual impairment and blindness in Europe and their prevention. Br J Ophthalmol.

[14] Congdon N, O'Colmain B, Klaver CCW, Klein R, Muñoz B, Friedman DS (2004). Causes and prevalence of visual impairment among adults in the United States. Arch Ophthalmol.

[15] Hadaegh F, Bozorgmanesh MR, Ghasemi A, Harati H, Saadat N, Azizi F (2008). High prevalence of undiagnosed diabetes and abnormal glucose tolerance in the Iranian urban population: Tehran Lipid and Glucose Study. BMC Public Health.

[16] Javadi MA, Katibeh M, Rafati N, Dehghan MH, Zayeri F, Yaseri M (2009). Prevalence of diabetic retinopathy in Tehran province: a population-based study. BMC Ophthalmol.

[17] Statistical Centre of Iran. Population and Housing Census 2011.Available at:http:// www.amar.org.ir.

[18] Mehrdad R (2009). Health System in Iran. Japan Medical Assoc J.

[19] World Health Organization (2007). Everybody business: strengthening health systems to improve health outcomes-WHO's framework for action.

[20] Blancht K, Gilbert C, Lindfield R (Eds): *Eye Health Systems Assessment (EHSA): How to Connect Eye Care with the General Health.* London: International Centre for Eye Health (ICEH); 2012.

[21] World Health Organization (2005). State of the World’s Sight Vision 2020: the Right to Sight 1999–2005.

[22] Li Z, Cui H, Zhang L, Liu P, Yang H (2009). Cataract blindness and surgery among the elderly in rural southern Harbin China. Ophthalmic Epidemiol.

[23] Murthy GV, Ellwein LB, Gupta S, Tanikachalam K, Ray M, Dada VK (2001). A population-based eye survey of older adults in a rural district of Rajasthan: II. Outcomes of cataract surgery. Ophthalmology.

[24] Neena J, Rachel J, Praveen V, Murthy GV (2008). Rapid Assessment of Avoidable Blindness in India. PLoS One.

[25] Khumbo Kalua1, Francis Masiye3, Vincent Jumbe3,4, John Barrows5, Victoria Sheffield5. Finding community solutions to improve access and acceptance of cataract surgery, optical correction and follow up in children in Malawi. Health 2013;**5**:DOI:10.4236/health.2013.510208.

[26] Edson Eliah SL, Khumbo K, Paul C, Michael G, Ken B. Task shifting for cataract surgery in eastern Africa: productivity and attrition of non-physician cataract surgeons in Kenya, Malawi and Tanzania. Hum Resour Health. 2014;12 Suppl 1:S4. 10.1186/1478-4491-12-S1-S4 PMC4108920 25859627

[27] Katibeh M, Ziaei H, Rajavi Z, Hosseini S, Javadi M-A. Profile of cataract surgery in Varamin Iran: a population based study. Clin Experiment Ophthalmol. 2013:doi: 10.1111/ceo.12185. [Epub ahead of print]. 10.1111/ceo.12185 23927430

[28] Hashemi H, Alipour F, Mehravaran S, Rezvan F, Fotouhi A, Alaedini F (2009). Five year cataract surgical rate in Iran. Optom Vis Sci.

[29] World Health Organization (2007). Global Initiative for the Elimination of Avoidable Blindness : action plan 2006–2011.

[30] Palmer JJ, Chinanayi F, Gilbert A, Pillay D, Fox S, Jaggernath J (2014). Mapping human resources for eye health in 21 countries of sub-Saharan Africa: current progress towards VISION 2020. Hum Resour Health.

[31] Palmer JJ, Chinanayi F, Gilbert A, Pillay D, Fox S, Jaggernath J (2014). Trends and implications for achieving VISION 2020 human resources for eye health targets in 16 countries of sub-Saharan Africa by the year 2020. Hum Resour Health.

[32] Eze BI, Maduka-Okafor FC (2009). An assessment of the eye care workforce in Enugu State, south-eastern Nigeria. Human Resources Health.

[33] Habtamu E, Eshete Z, Burton MJ (2013). Cataract surgery in Southern Ethiopia: distribution, rates and determinants of service provision. BMC Health Serv Res.

[34] Dolea C, Stormont L, Braichet JM (2010). Evaluated strategies to increase attraction and retention of health workers in remote and rural areas. Bull World Health Organ.

[35] du Toit R, Brian G (2009). Mid-level cadre providing eye care in the context of Vision 2020. N Z Med J.

[36] Murthy GVS, Gupta SK, Bachani D, Tewari HK, John N (2004). Human resources and infrastructure for eye care in India: current status. Natl Med J India.

[37] Blanchet K, Patel D (2012). Applying principles of health system strengthening to eye care. Indian J Ophthalmol.

[38] Odusote K (2005). People deliver eye care: managing human resources. Community Eye Health.

[39] Razavi H, Kuper H, Rezvan F, Amelie K, Mahboobi-Pur H, Oladi MR (2010). Prevalence and causes of severe visual impairment and blindness among children in the lorestan province of iran, using the key informant method. Ophthalmic Epidemiol.

[40] Lewallen S, Roberts H, Hall A, Onyange R, Temba M, Banzi J (2005). Increasing cataract surgery to meet Vision 2020 targets; experience from two rural programmes in east Africa. Br J Ophthalmol.

[41] The World Bank (2008). Islamic Republic of Iran-Health Sector Review- Volume I. Main Report.

